# Adsorption of Mercury in Aqueous Solutions by Functionalized Cellulose Extracted from Soybean Hulls

**DOI:** 10.1002/cplu.202400707

**Published:** 2025-04-09

**Authors:** Monica Rigoletto, María Rapp, Amaya Arencibia, María‐José López‐Muñoz, Maria Laura Tummino, Nieves Fernández de Paz, Enzo Laurenti

**Affiliations:** ^1^ Department of Chemistry University of Turin Via P. Giuria 7 10125 Torino Italy; ^2^ Departamento de Tecnología Química Energética y Mecánica ESCET Universidad Rey Juan Carlos C/ Tulipán s/n 28933 Móstoles Spain; ^3^ Departamento de Tecnología Química y Ambiental ESCET Universidad Rey Juan Carlos C/ Tulipán s/n 28933 Móstoles Spain; ^4^ Instituto de Tecnologías para la Sostenibilidad Universidad Rey Juan Carlos C/ Tulipán s/n 28933 Móstoles Spain; ^5^ Istituto di Sistemi e Tecnologie Industriali Intelligenti per il Manifatturiero Avanzato (STIIMA) Consiglio Nazionale delle Ricerche (Cnr) Corso G. Pella 16 13900 Biella Italy; ^6^ Metrohm Hispania C/ Aguacate 15 28044 Madrid Spain

**Keywords:** adsorption, aquaculture, celluloses, mercury, sustainable chemistry

## Abstract

The presence of potentially toxic elements (PTEs) in drinking water and the food chain is a well‐known hazard to human health. Among PTEs, mercury is particularly dangerous for humans and other living organisms due to its wider effects on internal organs. Hg contamination is a critical issue for water bodies used for aquaculture, making its elimination mandatory. Among the techniques proposed for Hg removal, adsorption is advantageous because of its versatility, absence of secondary pollution, and relatively low cost, especially when adsorbents can be obtained from waste materials. In this article, adsorbent materials are synthesized by introducing thiols and primary amino groups into cellulose fibers isolated from soybean hulls. After characterization, the ability of the materials to remove mercury from both ultrapure and aquaculture water solutions is tested. The results confirm the affinity of Hg for thiol groups, leading to the adsorption of 44 mg(Hg)/g in a wide pH range. The amino‐modified material adsorbs ≈50% Hg less than the thiol‐functionalized one. Test in real water shows that organic matter and salts influence the Hg adsorption process, without affecting the overall efficiency. Finally, in real water, a final concentration below the Hg legal limit for human consumption (1 μg L^−1^) is found.

## Graphical abstract

Reuse of agricultural waste as a source of adsorbent cellulose‐based materials is a low‐cost and sustainable approach to remove potentially toxic elements (PTEs) from aqueous solutions. This approach is successfully applied in the removal of mercury from aquaculture water, an important source of food where the absence of PTEs is a fundamental requisite for safety of humans and survival of ecosystems.

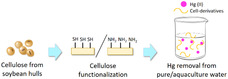

## 1 Introduction

The preservation of water quality is a fundamental duty because the socioeconomic well‐being and livelihood of the human population, as well as the survival of ecosystems and biodiversity, depend on it. The continuous population growth and climate changes contribute to the water crisis, which leads to the death of more than 13,000 people every year due to the lack of water resources availability and water contamination.^[^ ^1^ ^]^ However, this represents a huge challenge for scientists as the development of industrial activities and ever‐increasing urbanization represent some of the main sources of environmental pollution.

Among water pollutants, potentially toxic elements (PTEs) represent a well‐known danger to human health and biota safety in general due to their toxic effects even at very low concentrations.^[^ ^2^ ^]^ For these reasons, they are subject to regulations that govern their use and limit their release into the environment. Despite this, there are many industrial activities that produce PTEs containing waste that, if not treated properly, could release these pollutants into the environment where they cannot be degraded, thereby giving rise to bioaccumulation and biomagnification processes.^[^ ^3^, ^4^ ^]^

Among the PTEs, mercury (Hg) has received special attention due to its high toxicity.^[^ ^5^ ^]^ It is released into the environment from different anthropogenic sources, including iron, steel, cement, and gold industries; nonferrous metal smelting; chloro‐alkali industries; and direct mercury production industries.^[^ ^6^ ^]^

Mercury and its compounds, both organic and inorganic, have neurotoxic, genotoxic, teratogenic, carcinogenic, and bioaccumulative effects.^[^ ^7^, ^8^ ^]^ Therefore, the Hg concentration limit in drinking water is set at very low concentration by different institutions. According to the World Health Organization (WHO),^[^ ^9^ ^]^ the US Environmental Protection Agency (US EPA)^[^ ^10^ ^]^ and the European Directive (EU) 2020/2184, the Hg concentration limit in drinking water is 1 μg L^−1^. Other restrictions are, in turn, oriented to minimize the mercury content in food, especially within fishery products, as reported in the EU Commission Regulation 2023/915 of 25 April 2023.^[^ ^11^ ^]^

In recent years, different remediation techniques have been developed to reach this low concentration limit and, among them, adsorption is considered one of the most effective methods for the purification of heavy metal‐polluted water.^[^ ^12^ ^]^ Several carbon‐based materials, such as activated carbon, biochar or MOF,^[^ ^13^ ^]^ mesoporous silica,^[^ ^14^ ^]^ or magnetic particles,^[^ ^15^ ^]^ have been proposed in the literature as typical adsorbents for heavy metals removal from aqueous media, although they could be expensive in some cases.^[^ ^16^ ^]^ In the last decades, biopolymer‐based materials have been reported as an interesting alternative to classic adsorbents due to their low cost, high availability, ease of use and chemical modification, high biodegradability, and minimal toxicity.^[^ ^17^ ^]^

Since the circular economy concept has been included in European regulations and action plans that promote a zero‐waste economy model,^[^ ^18^ ^]^ biopolymers’ feedstock choice increasingly falls on agroindustrial and food wastes, which are rich in biomasses. These contain high amounts of cellulose, starch, alginate and other plant‐based biopolymers exploitable for a wide range of applications.^[^ ^19^, ^20^, ^21^ ^]^ Several biopolymer extraction strategies have been reported in the literature, from the most traditional methods, that is, chemical extraction with basis or acids,^[^ ^22^ ^]^ deep eutectics solvents,^[^ ^23^, ^24^ ^]^ and enzymatic or bacterial extraction,^[^ ^25^ ^]^ to the latest techniques, such as microwave and ultrasound‐assisted extraction.^[^ ^26^ ^]^ Furthermore, various functionalization techniques have been developed for to introduce different chemical groups in the polymeric matrix in order to improve their exploitability.

It is well known that mercury shows a high affinity for functional groups containing sulphur and nitrogen in agreement with the hard‐soft acid‐base theory.^[^ ^27^, ^28^, ^29^ ^]^ Indeed, Lewis acids like Hg(II) could coordinate with lone pairs of Lewis bases (like N and S atoms), forming stable coordination complexes.^[^ ^29^ ^]^ Commercial and extracted cellulose was also modified with different techniques to increase its adsorption properties toward Lewis acids.^[^ ^8^, ^30^, ^31^ ^]^

Considering this framework, in this research work, we coupled water remediation challenges with the circular economy approach: adsorbent materials were obtained by functionalizing waste‐deriving cellulose with thiols and primary amino groups. The resulting materials (namely Cell‐SH and Cell‐NH_2_) were characterized by several techniques and their ability to remove mercury from both ultrapure and real water‐spiked solutions was deeply investigated to evaluate a concrete application in real contexts.

## 2 Results and Discussion

### 2.1 Characterization of the Functionalized Cellulose Samples

Pristine soybean‐derived cellulose and amino‐containing samples have been deeply characterized in a previous work^[^ ^32^ ^]^ in which the composition, morphology, crystallinity, and thermal behavior were studied. In the present work, we completed the characterization of Cell‐SH in comparison with the other two materials.

#### 2.1.1 Elemental Analysis

The elemental composition (C, H, N, S) of cellulose, Cell‐SH and Cell‐NH_2_, expressed in mass weight percentage and in mmol g^−1^, is shown in **Table** **1**. The results confirm the success of both functionalization processes despite their different outcomes: 15.5% w/w of sulfur and 0.48% w/w of nitrogen were introduced in Cell‐SH and Cell‐NH_2_, respectively. Consequently, the estimated amount of grafted silane is 4.83 mmol g^−1^ for Cell‐SH and 0.346 mmol/g for Cell‐NH_2_. The value for amino‐functionalized cellulose is in total agreement with that obtained by Tummino et al.^[^ ^32^ ^]^

**Table 1:** Elemental composition of pristine waste‐derived cellulose and functionalized samples.

	**Cellulose**	**Cell‐SH**	**Cell‐NH**_**2**_
C [% w/w]	41.9 ± 0.09	42.3 ± 1.1	30.4 ± 0.4
C [mmol g^−1^]	34.9 ± 0.08	35.2 ± 0.3	25.8 ± 0.9
H [% w/w]	5.90 ± 0.07	6.07 ± 0.07	5.42 ± 0.09
H [mmol g^−1^]	58.4 ± 0.7	60.2 ± 0.9	53.7 ± 0.7
N [% w/w]	–	–	0.48 ± 0.04
N [mmol g^−1^]	–	–	0.346 ± 0.026
S [% w/w]	–	155 ± 2.3	–
S [mmol g^−1^]	–	4.8 ± 0.7	–

#### 2.1.2 ATR‐FTIR Spectroscopy

**Figure 1:**
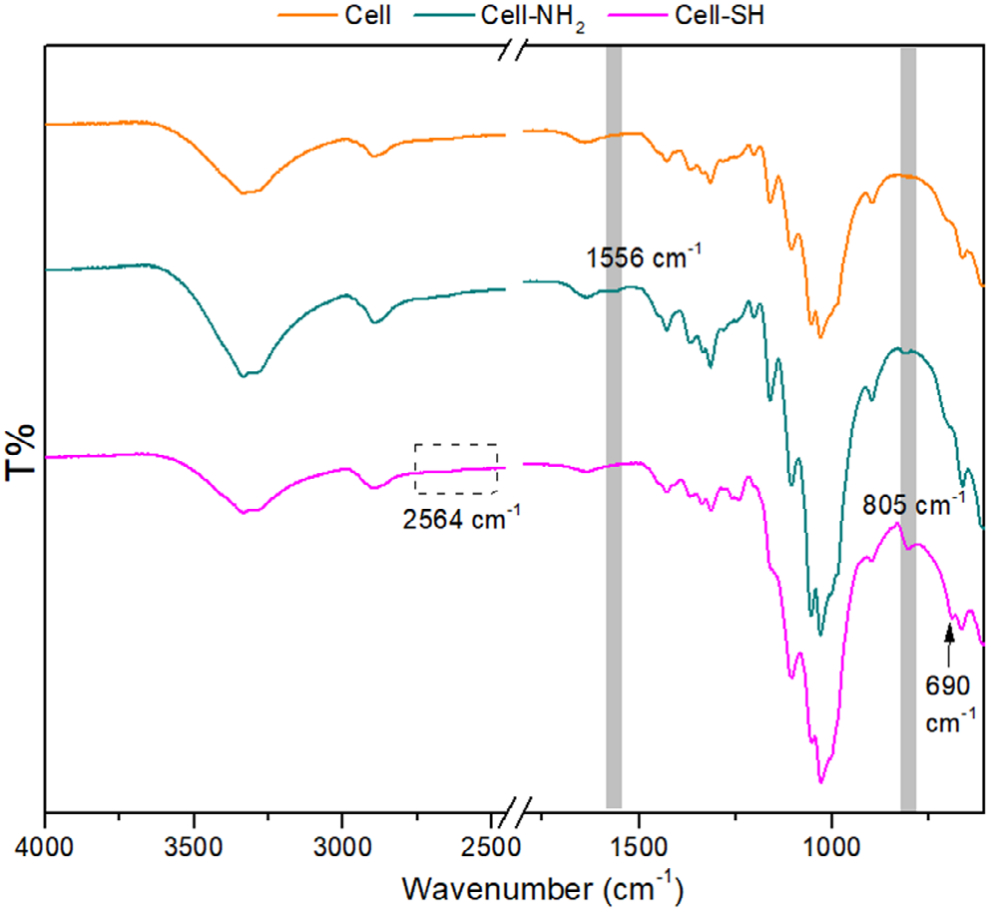
ATR‐FTIR spectra of pristine hull‐derived cellulose; Cell‐NH_2_ sample and Cell‐SH sample.

The insertion of nitrogen and sulfur in the functionalized materials was also confirmed by the attenuated total reflectance Fourier transform infrared (ATR‐FTIR) spectra, as shown in **Figure** **1**.

All the samples present the characteristic pattern of cellulose‐based materials. The signals between 3400 and 3200 cm^−1^ are related to the stretching vibration of —OH groups,^[^ ^33^ ^]^ whereas the asymmetric and symmetric stretching vibrations of C—H contained both in the cellulose structure and in the side chains of APTES and MPTMS are visible between 3000 and 2800 cm^−1^; the absorption band assigned to the bending vibration of adsorbed water could be found at 1640 cm^−1^ while the characteristic signals between 1430–1314 cm^−1^ are attributable to the symmetric bending of CH_2_, and the bending vibration of C—O and C—H in the polysaccharide ring. The 1160–1030 cm^−1^ signals are related to the C—O stretching and the C—H rocking vibrations of the pyranose ring skeleton, while the β‐glycosidic linkage signal appears at 896 cm^−1^.^[^ ^34^, ^35^, ^36^ ^]^

In both ATR‐IR spectra of Cell‐NH_2_ and Cell‐SH, a signal appears at about 800 cm^−1^, which can be correlated to the Si—O—Si bending due to the introduction and polymerization of silanizing agents (i.e., APTES and MPTMS).^[^ ^37^, ^38^ ^]^ Furthermore, as additional evidence of the functionalization, in the Cell‐NH_2_ spectrum appears a band at 1556 cm^−1^ that can be ascribed to the N—H bending vibration in primary amines.^[^ ^39^, ^40^ ^]^

On the other hand, in the Cell‐SH spectrum, there are further signals that can be related to the introduction of the silanizing reagent. Indeed, according to the literature, the weak bands at 1260–1240 cm^−1^ could be assigned to silicon–carbon vibration modes,^[^ ^41^, ^42^, ^43^ ^]^ whereas the peak at 690 cm^−1^ indicates the presence of C—S linkages (stretching vibration).^[^ ^44^, ^45^ ^]^ Although sometimes a very weak band at 2564 cm^−1^ attributable to the S—H stretching mode^[^ ^30^ ^]^ could also be detected in the Cell‐SH spectrum, the presented analysis did not let it emerge.

#### 2.1.3 Morphological Analysis and Microanalysis

The morphological properties of both modified cellulose samples were analyzed by electron microscopy.

**Figure 2:**
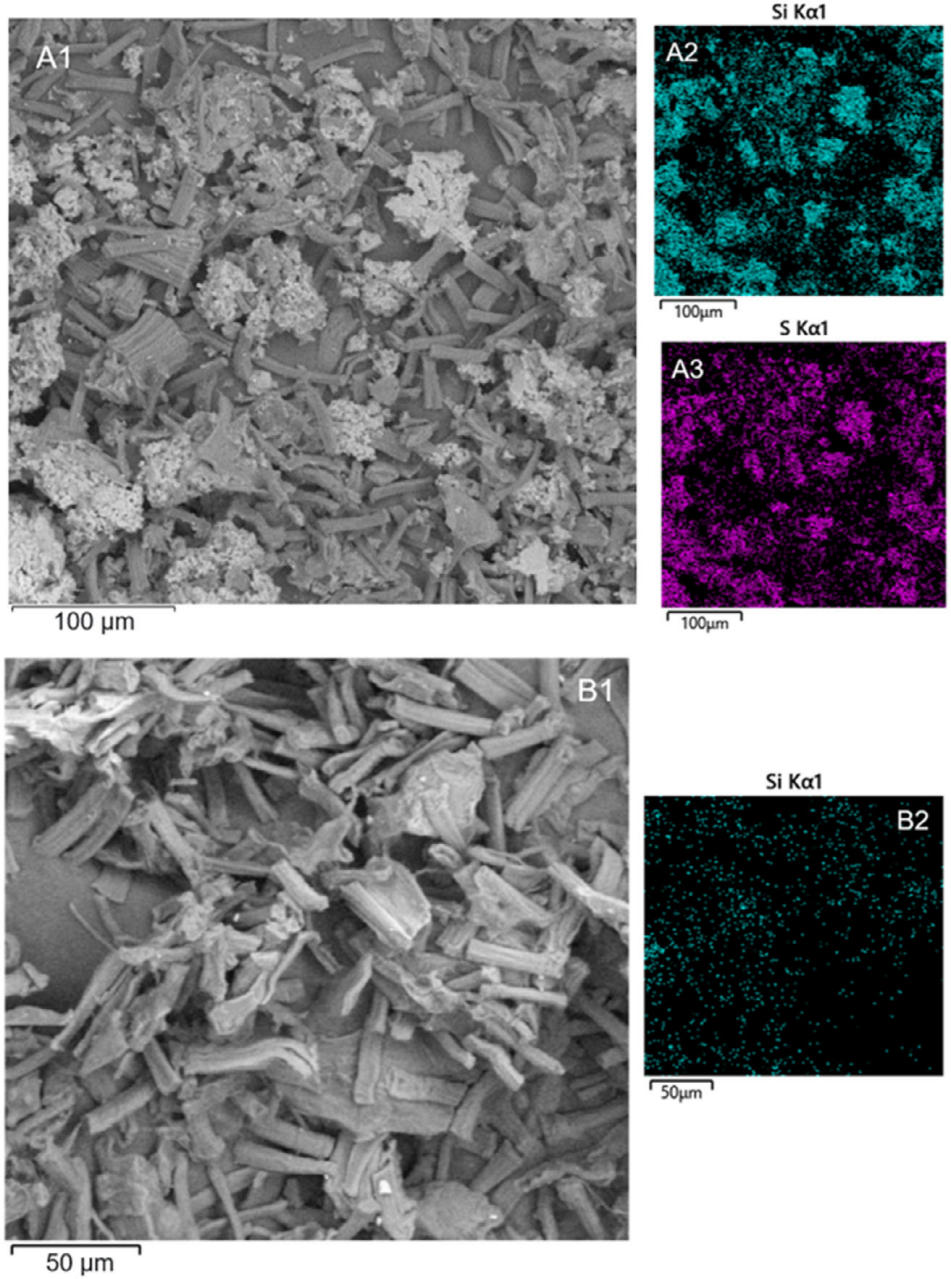
FE‐SEM images. A1) Cell‐SH image; A2) surface distribution of Si in Cell‐SH sample; A3) surface distribution of S in Cell‐SH sample; B1) Cell‐NH_2_ image; B2) surface distribution of Si in Cell‐NH_2_ sample.

According to Tummino et al.^[^ ^32^ ^]^ soybean hull‐derived cellulose shows a fibrous morphology characterized by a fiber length of around 35 μm and the sporadic presence of cemented regions related to impurities or residues of hemicellulose and lignin. As shown in **Figure** **2**A1,B1, no significant morphological changes were induced on cellulose fibers by MPTMS and APTES functionalization, although dispersed aggregates are evidenced in Cell‐SH.

The surface distribution of sulfur and nitrogen in the cellulosic matrix was investigated using field emission‐scanning electron microscopy (FE‐SEM) analysis. As shown in Figure 2A3, S‐atoms seem to form aggregates on the Cell‐SH fibers and a similar distribution is also confirmed for Si‐atoms (Figure 2A2), which are present in a ratio 1:1 with S, since both elements were simultaneously introduced in the material through MPTMS‐functionalization. The aggregation of MPTMS derivative could be related to a homopolycondensation of alkoxysilanes leading to the formation of a polysiloxane layer.^[^ ^41^ ^]^

On the other hand, in the Cell‐NH_2_ sample, the N‐atom distribution is not visible, probably due to the lower amount introduced in the matrix compared to sulfur. However, the distribution map of Si in this sample (Figure 2B2) suggests a good dispersion of APTES‐derived functional groups on the Cell‐NH_2_ fibers, leading to the assumption of similar behavior for nitrogen distribution.

#### 2.1.4 Thermal Analysis

**Figure 3:**
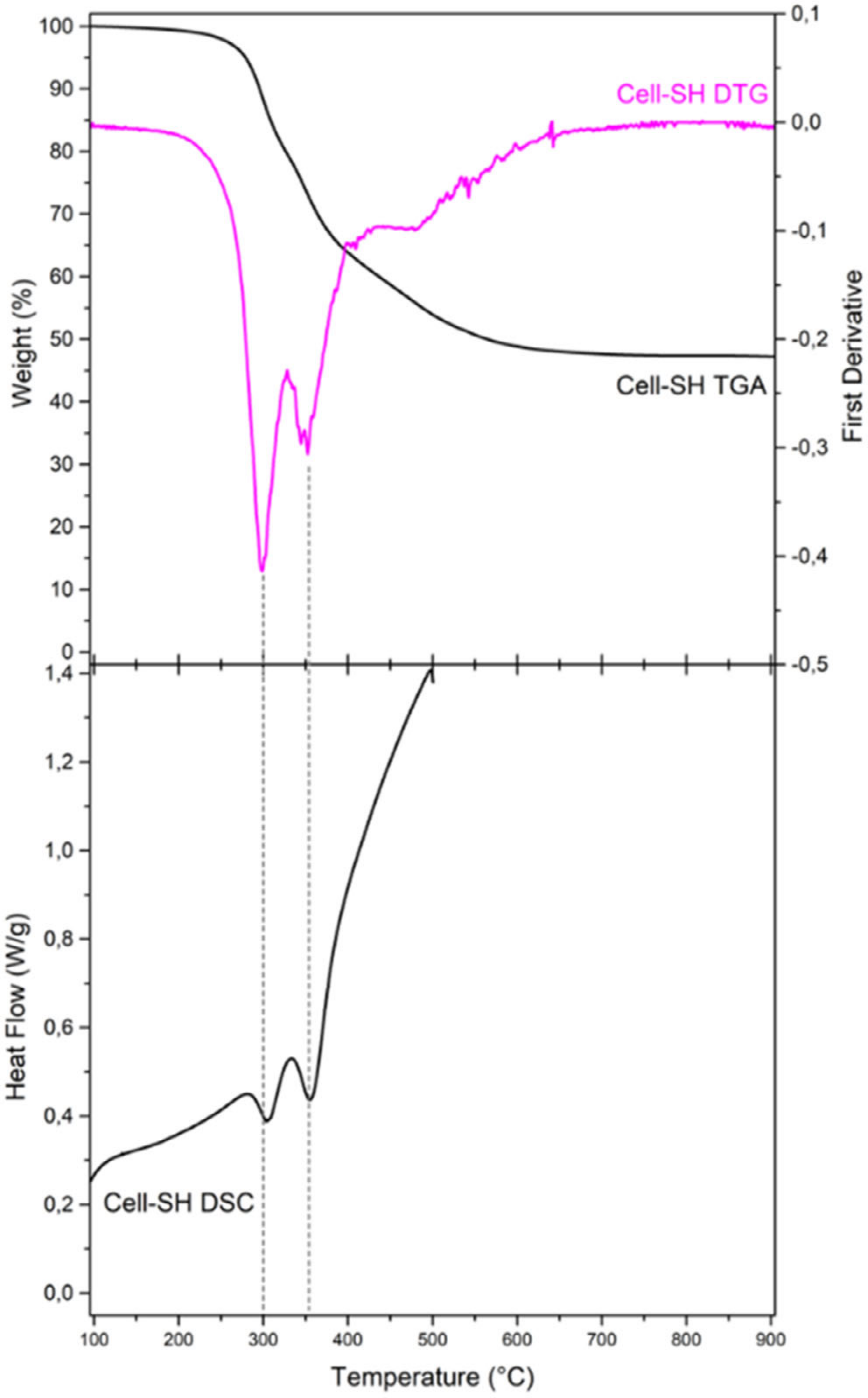
Cell‐SH thermal analysis. Top: TGA and DTG curves. Bottom: DSC curve.

**Figure** **3** shows Cell‐SH outcomes of thermogravimetric analysis (TGA), derivative thermogravimetry (DTG), and differential scanning calorimetry (DSC) analysis. As evidenced by the TGA thermogram, the remaining residue at 900 °C is 47%, which is higher than the amounts previously found for cellulose (12%) and Cell‐NH_2_ (21%).^[^ ^32^ ^]^ The charred residue, which is related to the formation of both pyrolyzed cellulose and silicon oxycarbide, can be correlated to the grafted silane amount.^[^ ^46^ ^]^ The highest residual weight (%) of the Cell‐SH is in agreement with the data obtained from the FE‐SEM‐(EDS) energy dispersive spectroscopy and the elemental analysis that evidence a greater content of MPTMS‐deriving functionalities. Similar results have been obtained by Thimmiah and Nallathambi, who observed a TGA residue of around 40% for cellulose extracted from *Aloe vera* and functionalized with MPTMS, with respect to 14% found in the raw fibers.^[^ ^47^ ^]^

Furthermore, DTG and DSC curves suggest a different pyrolytic pathway for the sulfur‐containing sample with respect to those observed for nitrogen‐containing and pristine celluloses. Indeed, two temperatures of maximum weight loss percentage can be identified in the DTG graph (300 and 355 °C), which correspond to the endothermic peaks in DSC measurements, and an additional smaller peak at 500 °C. Cell and Cell‐NH_2_ DSC curves showed only the cellulose characteristic endothermic peak centered at 350–360 °C^[^ ^32^ ^]^ due to a one‐step decomposition. The different decomposition stages experienced by Cell‐SH could be related to the presence of a surface layer of condensed polysiloxanes that decompose in two steps at 350–450 °C and 450–550 °C.^[^ ^41^ ^]^

#### 2.1.5 N_*2*_ *Adsorption‐Desorption Isotherms*

The textural properties were investigated through the analysis of nitrogen adsorption–desorption isotherms at 77 K of modified and nonmodified cellulose samples.

**Figure 4:**
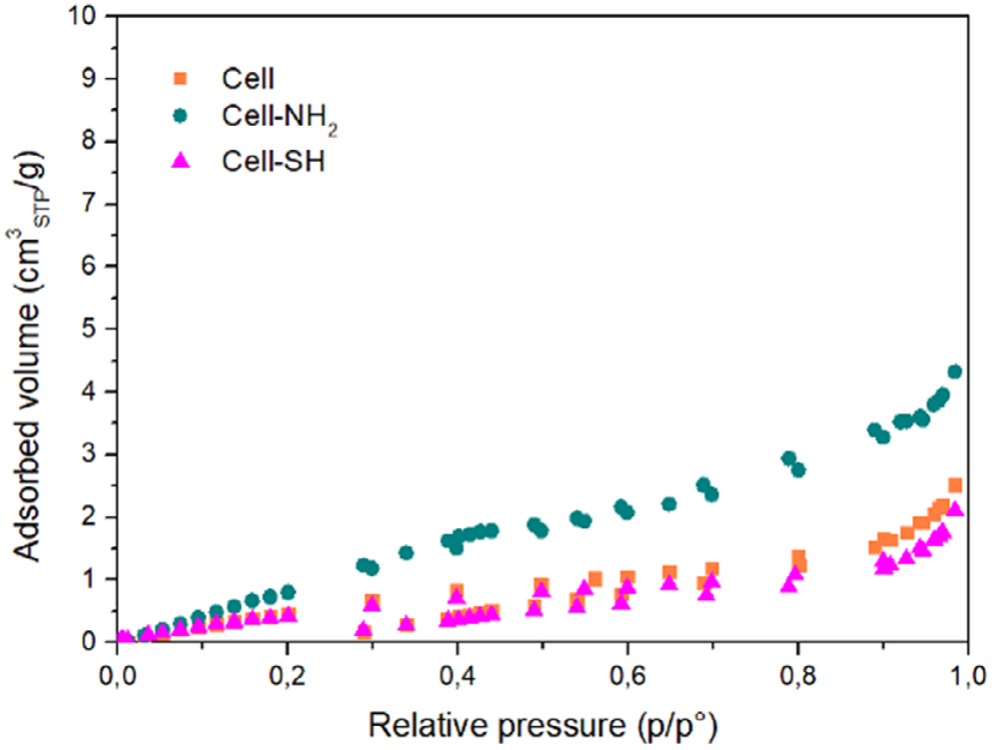
N_2_ adsorption–desorption isotherms at 77 K of synthesized samples (BET analysis).

The three materials were found to be nonporous with a measured pore volume of 0.03, 0.06, and 0.03 m^3^ g^−1^ for cellulose, Cell‐NH_2_, and Cell‐SH, respectively. As shown in **Figure** **4**, they are characterized by type II adsorption isotherms of IUPAC classification and BET surface area values around 3, 2, and 7 m^2^ g^−1^ for cellulose, Cell‐SH, and Cell‐NH_2_ samples, respectively. These results are in agreement with Bismarck et al.^[^ ^48^ ^]^ who reported that natural fibers are generally nonporous solids with very low specific surface area, which could slightly increase in dried swollen fibers.

#### 2.1.6 Surface Charge Analysis

The point of zero charge (PZC) was determined for all the cellulose‐based samples in water suspension.

**Figure 5:**
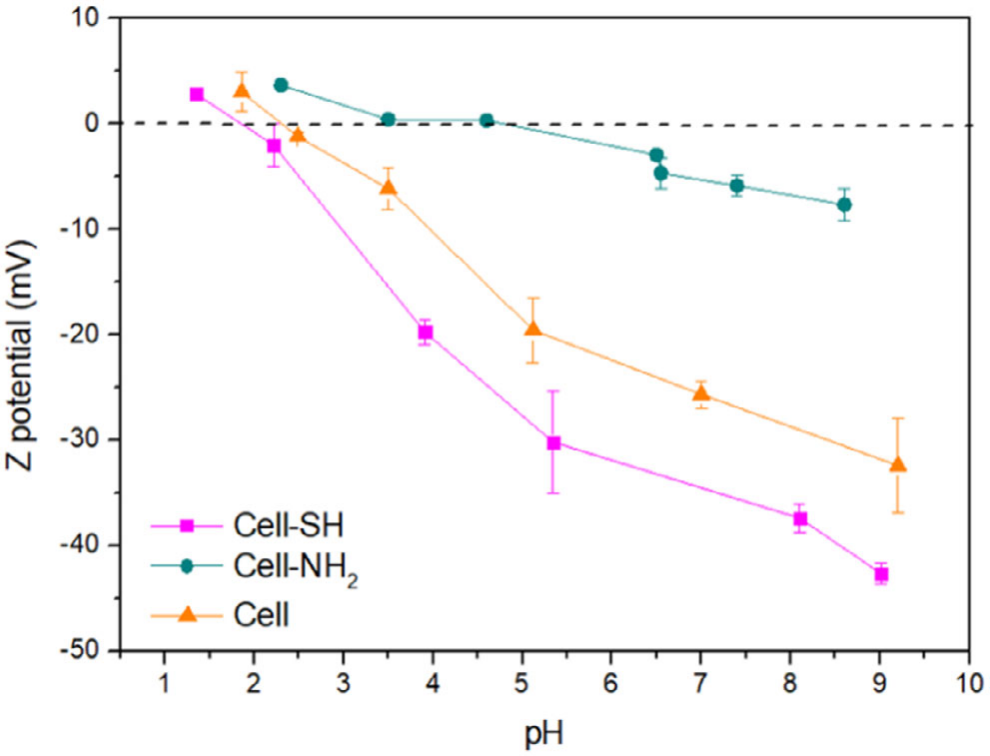
Z‐potential of all samples in aqueous suspensions as a function of pH.

**Figure** **5** displays the Z‐potential values as a function of pH. The low PZC of cellulose and Cell‐SH is related to the presence of —OH and —SH surface groups that lead to a negative surface charge for most of the pH range studied. Increasing the acidity of the suspension decreases the magnitude of zeta potential due to the repression of the deprotonation of the functional groups. At pH values lower than the PZC, the zeta potential becomes positive as a result of protonation.^[^ ^49^ ^]^ Both cellulose and Cell‐SH show a linear dependence of zeta potential on increasing pH, reaching −32 and −43 mV, respectively, at pH 9, meaning that the suspensions become more stable by decreasing the acidity of the medium. Indeed, Z‐potential is also considered a measure of suspension electrostatic stability: for absolute values above 30 mV, the repulsive forces prevail and no aggregation phenomena occur.^[^ ^50^ ^]^

Nonfunctionalized cellulose shows slightly lower PZC than that reported in the literature (3.7)^[^ ^49^, ^51^ ^]^ and a greater slope of the curve describing the trend of the Z‐potential as a function of pH. This could be probably due to some residual functionalities deriving from the cellulose isolation treatment.

The Cell‐SH Z‐potential trend is also in agreement with different studies that reported similar results in terms of absolute values in the whole pH range.^[^ ^52^, ^53^ ^]^

On the other hand, according to the literature,^[^ ^54^ ^]^ the introduction of primary amines shifts the isoelectric point of the Cell‐NH_2_ sample toward a more alkaline pH (5.0). In this case, the suspension does not reach stability conditions, maintaining some aggregation tendency in the studied pH range since the maximum absolute value of Z potential reached is −8 mV.^[^ ^50^ ^]^

### 2.2 Characterization of Aquaculture Water Samples

Before using aquaculture water as the matrix for the Hg(II) adsorption tests, the sample was analyzed using different techniques. As summarized in **Table** **2**, it was characterized by a pH of 7.3, a hardness value of 102.74 mg L^−1^ of CaCO_3_, and 28.54 mg L^−1^ of total organic carbon. The heavy metals and semimetals content (As, Cd, Cu, Pb, and Hg) was below the detection limit (LOD) and anions’ concentrations were near the value indicated in the Directive (EU) 2020/2184, meaning that the selected matrix was not polluted. Therefore, for the Hg(II) adsorption tests, the stock solution was prepared without any concentration correction.

**Table 2:** Real water sample characterization and comparison with the limit concentration values set by the European Directive.

Parameter	Unit	Value	Limits for human usagea)	Limits for protection of salmonidsd)
pH	–	7.3	6.5–9.5b)	6–9
Hardness	mg L^−1^ CaCO_3_	102.74	–	–
TOC	mg L^−1^	28.54	–	–
Ca	mg L^−1^	24.5	–	–
K	mg L^−1^	1.91	–	–
Mg	mg L^−1^	10.6	–	–
Na	mg L^−1^	5.06	200b)	–
Si	mg L^−1^	4.54	–	–
As	μg L^−1^	<LOD	10c)	50
Cd	μg L^−1^	<LOD	5c)	2.5
Cu	mg L^−1^	<LOD	2c)	0.04
Pb	μg L^−1^	<LOD	5c)	10
Hg	μg L^−1^	<LOD	1c)	0.5
F^−^	mg L^−1^	1.44	1.5c)	6
Cl^−^	mg L^−1^	24.7	250b)	–
NO_3_^−^	mg L^−1^	52.5	50c)	–
SO_4_^2−^	mg L^−1^	51.04	250b)	–
NO_2_^−^	mg L^−1^	0.634	0.5c)	0.01

a) Directive (EU) 2020/2184 of the European Parliament and of the Council of 16 December 2020 on the quality of water intended for human consumption, Annex I

b) Directive (EU) 2020/2184: indicator parameters

c) Directive (EU) 2020/2184: chemical parameters

d) (i) Directive (EU) 2006/44/EC of the European Parliament and of the Council of 6 September 2006 on quality of fresh waters needing protection or improvement to support fish life, Annex I; (ii) Italian Legislative Decree n. 152/2006 [Annex 2 and 5 part III], Section B.

### 2.3 Mercury Adsorption Test

#### 2.3.1 Adsorption Tests in Ultrapure Water

**Figure 6:**
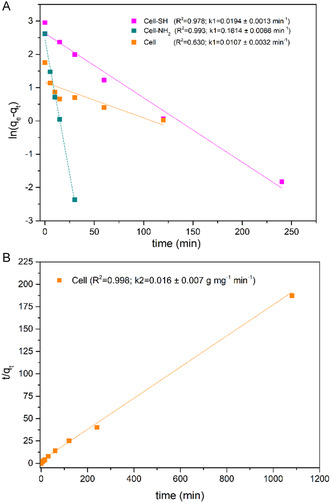
Linear fit of the kinetic data: A) pseudo‐first‐order model and B) pseudo‐second‐order model. Calculated kinetic constant values are reported in brackets.

The fitting of the adsorption kinetic profiles, obtained for the cellulose samples in contact with 20 mg L^−1^ of Hg(ii) as the initial concentration, is displayed in **Figure** **6**. It emerges that Cell‐SH and Cell‐NH_2_ behaviors fit well with a pseudo‐first‐order model, while for pristine cellulose a pseudo‐second‐order model seems more appropriate. All the adsorption data show a high initial rate, although the kinetic constant was found to be smaller for the Cell‐SH. Equilibrium conditions are reached in less than 5 h but a significantly different adsorption percentage can be observed among the various materials. Indeed, only the Cell‐SH sample is able to completely remove mercury from the initial solution, while a reduced adsorption extent is observed for Cell and Cell‐NH_2_ (29% and 78%, respectively). On the basis of these results and subsequent kinetic tests at a higher mercury concentration (100 mg L^−1^, data not shown), adsorption measurements were carried out in ultrapure water by analyzing the final concentrations after 18 h of contact between mercury‐containing solutions and the adsorbent materials.

The adsorption isotherms were obtained as described in the Experimental section.

**Figure 7:**
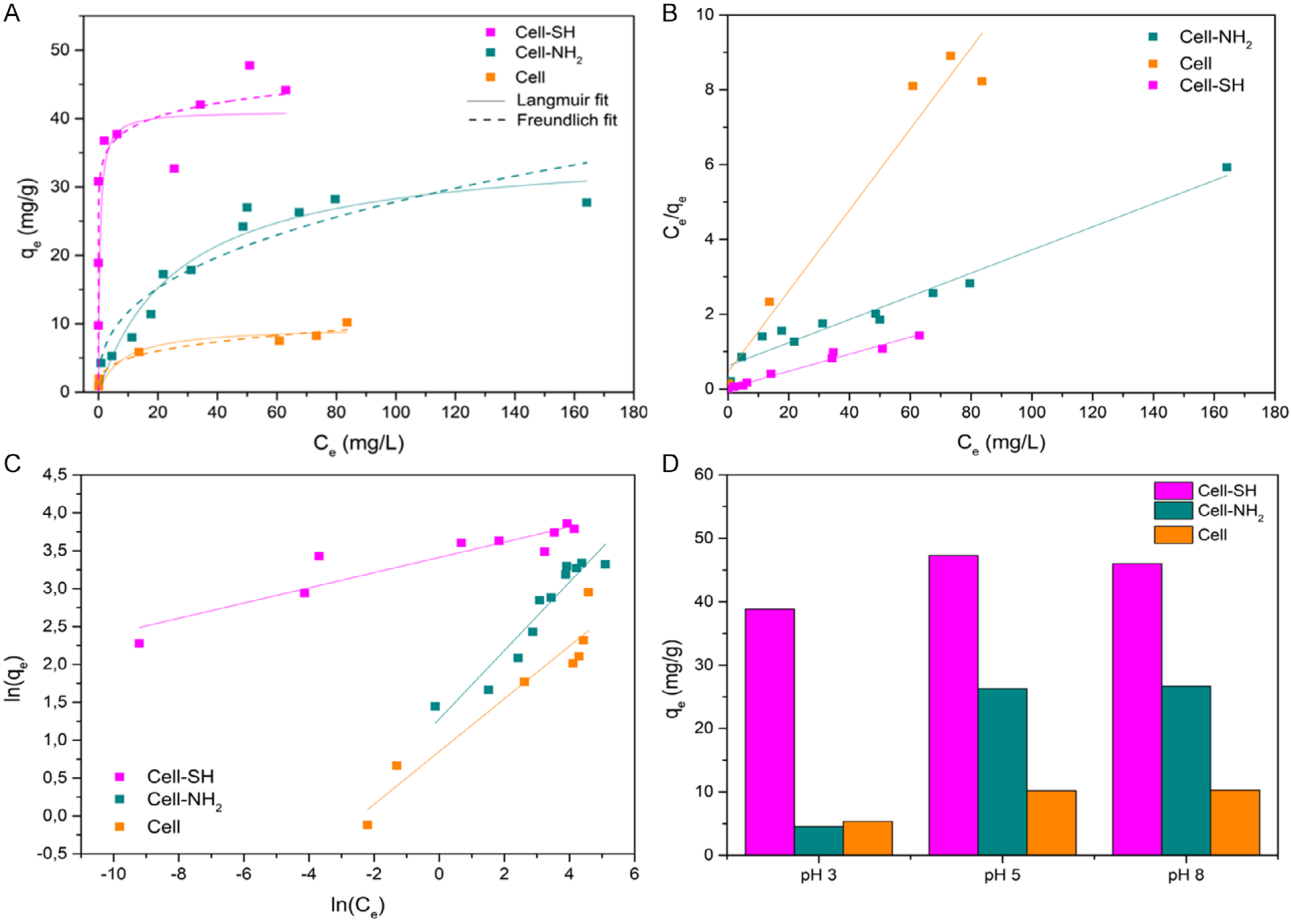
A) Experimental equilibrium data for mercury adsorption at pH 5, and Langmuir and Freundlich fit; B) Linear Langmuir model; C) Linear Freundlich model; D) q_e_ values obtained for a 100 mg L^−1^ Hg(II) solution at different pH.

**Figure** **7**A shows the amount of mercury adsorbed q_e_ (mg g^−1^) plotted as a function of the equilibrium Hg(II) concentration C_e_ (mg L^−1^). The isotherms can be classified as L2‐type according to the Giles classification,^[^ ^55^ ^]^ with a significant slope at low mercury concentrations and a saturation value when the Hg(II) concentration in the solution increases.

The linear Langmuir and Freundlich plots are presented in Figure 7B,C, while the estimated adsorption parameters are summarized in **Table** **3**. For all the tested adsorbents, the best fitting of experimental data was obtained with the Langmuir model, suggesting that the adsorption of Hg(II) is mainly attributable to a chemisorption characterized by the formation of a monolayer of the adsorbate on the cellulose surface. The separation factor *R*_L_ is less than one for all the materials, indicating that mercury adsorption is a favorable process.

**Table 3:** Adsorption isotherms parameters for Hg(II) adsorption on cellulose‐based materials at pH 5.

Langmuir parameters				
Material	*K*_L_ [L mg^−1^]	*q*_m_ [mg g^−1^]	*R* ^2^	*R*_L_
Cell	0.24	9.23	0.961	0.31
Cell‐SH	1.1	43.86	0.982	0.02
Cell‐NH_2_	0.05	32.26	0.963	0.38

Freundlich parameters				
Material	*K*_F_ [L mg^−1^]	*n*	*R* ^2^	1/n
Cell	2.34	2.87	0.924	0.348
Cell‐SH	30.3	9.96	0.863	0.100
Cell‐NH_2_	3.59	2.21	0.909	0.452

As evidenced by the maximum adsorption capacity at pH ≈ 5, which is 9.23, 32.26, and 43.86 mg(Hg) g^−1^ for Cellulose, Cell‐NH_2_, and Cell‐SH, respectively, the introduction of thiols in the cellulose matrix ensures a greater removal of mercury. This is also highlighted by the *K*_L_ value, which expresses the affinity between adsorbate and adsorbent surface, and which is significantly higher for the Cell‐SH sample (1.1 L mg^−1^).

Indeed, the related isotherm shows a marked initial slope indicating that Cell‐SH has very high effectiveness at low levels of initial mercury concentrations: a value of *q*_e_ = 0.99 mg g^−1^ was estimated for Cell‐SH at the initial Hg(II) concentration of 1 mg L^−1^ and equilibrium concentration near zero. This behavior can be attributed to the strong affinity of sulfur toward mercury according to the principle of hard and soft acids and bases of Pearson.^[^ ^56^ ^]^

The effect of pH on the adsorption capacity of Cell‐SH, Cell‐NH_2_, and Cellulose was investigated as well. The *q*_e_ values obtained for a 100 mg L^−1^ Hg(II) solution at pH 3, 5, and 8 are shown in Figure 7D. As observed, increasing the pH from 5 to 8 did not lead to a significant difference in the adsorption capacities of the materials. However, by decreasing the pH to 3, a reduction in the amount of Hg(II) adsorbed by 18, 83, and 48% was observed for Cell‐SH, Cell‐NH_2_, and Cellulose, respectively. This behavior could be ascribed to two different factors: 1) the Z‐potential and surface charge of the three adsorbent materials as a function of pH, as discussed in paragraph 3.1.6 (Figure 3), and 2) the speciation of HgCl_2_. Indeed, at pH 3, HgCl_2_ is the dominant species in solution, whereas by increasing the pH value up to 5, a contribution of HgClOH has to be also considered. Moreover, at pH 8, the two species HgClOH and Hg(OH)_2_ coexist in the solution, becoming mercury hydroxide predominant at higher pH values.^[^ ^57^ ^]^

The variation in the maximum adsorption capacity as a function of pH could also be evidence of the different affinities between the functionalized cellulose surfaces and the characteristic mercury species at each pH value. According to the results obtained, Cell‐NH_2_ and pristine cellulose show a low affinity for HgCl_2_ but a high affinity for HgClO and Hg(OH)_2_. By contrast, Cell‐SH seems to be able to strongly interact with all the mercury species existing in the aqueous solution at different pH values.

Finally, it is reported in the literature that at low pH, metal adsorption could also be affected by the competition with protons for the occupation of the surface adsorption sites.^[^ ^58^ ^]^ This aspect could facilitate the removal of metal species adsorbed on the surface and allow the reuse of the material in subsequent adsorption–desorption cycles.

#### 2.3.2 Adsorption Tests in Spiked Real Water

Mercury adsorption tests were also carried out using the sampled aquaculture water as the aqueous matrix and Cell‐SH as the adsorbent. Since the natural content of Hg in the aquaculture water was under the detection limit (Table 2), the mercury stock solution was prepared as described in paragraph 2.6 without any concentration correction. The adsorption isotherm of mercury was obtained at 20 °C from many single experimental runs with the initial Hg(II) concentration ranging from 1 to 200 mg L^−1^. Moreover, the behavior of Cell‐SH was also compared with that of Cell and Cell‐NH_2_ at low (15 mg L^−1^) and high (100 mg L^−1^) initial Hg(II) concentrations.

**Figure 8:**
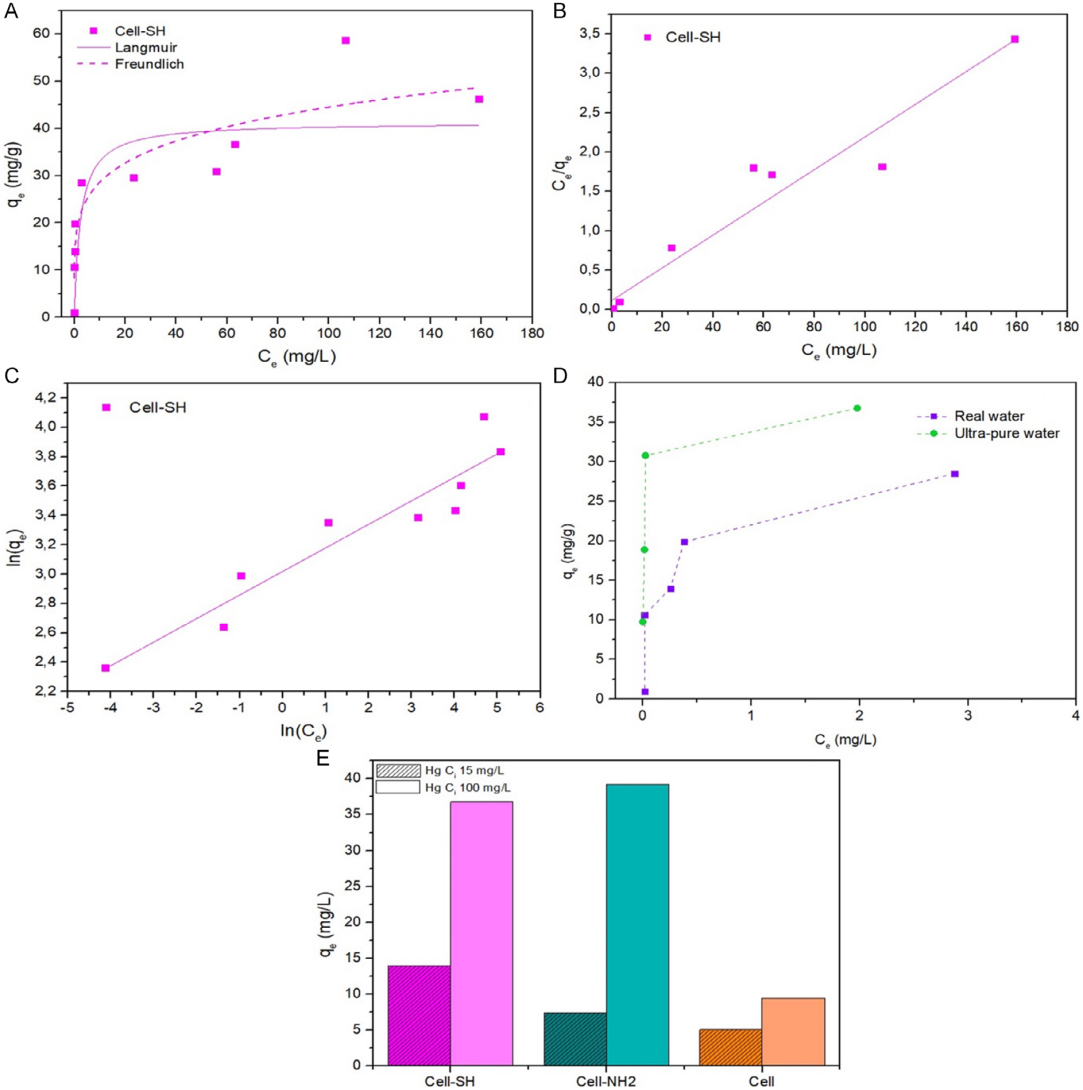
A) Experimental data for mercury adsorption in real water, and Langmuir and Freundlich fit. B) Linear Langmuir model. C) Linear Freundlich model. D) comparison between *q*_e_ of Cell‐SH in ultrapure water and real water at low Hg(II) concentration. E) *q*_e_ values obtained for a 15 mg L^−1^ and 100 mg L^−1^ mercury solution in real water matrix using cellulose, Cell‐NH_2_, and Cell‐SH.

Results are shown in **Figure** **8**A, where the amount of mercury adsorbed at equilibrium, *q*_e_, is plotted as a function of the resulting aqueous Hg(II) concentration, C_e_ (mg L^−1^). As observed in the previous experiments in ultrapure water, the isotherm can be classified as L2‐type according to the Giles classification.

The linear Langmuir and Freundlich plots are presented in Figure 8B,C, while the estimated adsorption parameters are summarized in **Table** **4**. As for the experiment in ultrapure water, the best fitting of experimental data was obtained with the Langmuir model, suggesting that the adsorption of Hg(II) is mainly attributable to a chemisorption characterized by the formation of a monolayer of the adsorbate on the cellulose surface. The separation factor R_L_ < 1 indicates that mercury adsorption is a favorable process.

**Table 4:** Adsorption isotherms parameters for Hg(II) adsorption in the spiked real water matrix.

Langmuir parameters				
Material	*K*_L_ [L mg^−1^]	*q*_m_ [mg g^−1^]	*R* ^2^	*R*_L_
Cell‐SH	0.17	48.3	0.936	0.1186

Freundlich parameters				
Material	*K*_F_ [L mg^−1^]	*n*	*R* ^2^	1/*n*
Cell‐SH	20.45	6.23	0.887	0.1604

Looking at the estimated maximum adsorption capacity *q*_m_, Cell‐SH shows a comparable performance in real water (48.3 mg g^−1^) and in ultrapure water (43.86 mg g^−1^), while the matrix effect is noticeable in the first part of the isotherm. Indeed, as shown in Figure 8D, for low Hg(II) initial concentration, the affinity of the Cell‐SH surface seems to be affected by the substances present in aquaculture water.

One of the factors affecting the results in real matrices could be represented by the dissolved organic matter since it shows complexing capabilities toward metals due to the presence of different functional groups. The complexation of mercury could hinder the reaching of the Cell‐SH surface, affecting the adsorption process.^[^ ^59^, ^60^, ^61^ ^]^ However, the relatively low TOC value (28.54 mg L^−1^) justifies a competitive effect at low Hg(II) concentration but without affecting the maximum adsorption capacity, resulting in a *q*_m_ comparable to that obtained in ultrapure water.

The presence of anions or cations could also affect the adsorbent capabilities of Cell‐SH. Indeed, according to recent studies on similar materials,^[^ ^8^, ^59^ ^]^ anions such as NO_3_^−^ do not affect mercury adsorption, SO_4_^2−^ shows a limited effect, while Cl^−^ more affects mercury removal due to its complexation capabilities that increase Hg(II) distribution in water.^[^ ^62^, ^63^ ^]^ The newly formed species HgCl_3_^−^ and HgCl_4_^2−^, which are negatively charged, could hardly interact with the surface of adsorbents with a strongly negative Z‐potential at the working pH, such as Cell‐SH (Figure 4).^[^ ^64^ ^]^

The stronger interaction of mercury with sulfur was also found in the presence of competing cations.^[^ ^65^, ^66^, ^67^ ^]^ Similar findings were reported by Wang et al. for amino‐functionalized materials, who tested the effect of typical wastewater background ions (Na^+^, Mg^2+^, Ca^2+^, K^+^) and other heavy metals (Co^2+^, Cu^2+^, Pb^2+^, Zn^2+^, Ni^2+^), confirming the anti‐interference ability of Hg(II) adsorption process.^[^ ^68^ ^]^

On the other hand, Cell‐NH_2_ adsorbent capacity does not change in the solution at low Hg(II) concentration, while it increases in the higher one. The improvement of the Cell‐NH_2_ performance and, thus, the flattening of the differences in adsorption efficiency between Cell‐NH_2_ and Cell‐SH in aquaculture water (Figure 8E) at 100 mg L^−1^ may be somehow attributed to the effect of the matrix.^[^ ^66^, ^69^, ^70^, ^71^ ^]^ One hypothesis is based on the fact that when ions are adsorbed via inner‐sphere association (namely through specific adsorption, such as ligand exchange), they are less susceptible to ionic strength, being able to even respond to it with greater adsorption.^[^ ^72^, ^73^ ^]^ However, the explanation that seems more reasonable, according to what has already been highlighted in ultrapure water experiments, is that Cell‐NH_2_ is more sensitive to mercury speciation and the formed species like HgCl_3_^−^ and HgCl_4_^2−^, although the overall slightly negative surface charge,^[^ ^74^ ^]^ can interact with amino groups.

Finally, additional tests were carried out with a 1 mg L^−1^ Hg(II) solution prepared in real water with different amounts of Cell‐SH as an adsorbent in order to reach a mercury concentration below the EU‐allowed limit for drinking water (1 μg L^−1^). The final concentrations of Hg(II) remaining in the solution containing 1, 2, and 3 mg mL^−1^ of Cell‐SH were 18, 11, and 0.8 μg L^−1^, respectively, indicating that the developed materials could be considered effective in the treatment of real mercury‐contaminated waters.

Overall, the Hg removal results obtained in this work fall within average values found in the literature for biomass‐derived adsorbents, except for differently functionalized microcrystalline cellulose,^[^ ^8^ ^]^ or activated carbons/biochars,^[^ ^75^ ^]^ in which generally favorable textural and/or porosity features play a major role.^[^ ^76^, ^77^ ^]^ A very recent review, indeed, summarizes the efficiencies of different mercury adsorbents, confirming our statement.^[^ ^78^ ^]^ Moreover, what comes to light from a broader examination of the studies focused on Hg decontamination is that many strategies rely on the presence of metal‐based compounds, often in the form of nanoparticles^[^ ^79^, ^80^ ^]^ that, although able to bring about high efficiencies, might be associated with secondary environmental side effects.^[^ ^81^, ^82^ ^]^ In the specific field of aquaculture, the presence of iron, aluminum, and zinc, not considered among the most dangerous metals and widely used in Hg adsorption,^[^ ^83^ ^]^ poses risks for the welfare of fish, potentially resulting in the reduction of oxygen transfer if precipitate salts.^[^ ^84^ ^]^

## 3 Conclusion

In this work, we synthesized two different cellulose‐based adsorbents, Cell‐NH_2_ and Cell‐SH, for mercury removal from polluted water by introducing both amino and thiol functionalities in a waste‐derived cellulose skeleton.

The outcomes of the synthesis revealed that Cell‐NH_2_ and Cell‐SH reached different degrees of functionalization, probably due to the different reagents involved (APTES and MPTMS) and the operating conditions. In particular, thiol groups were introduced in a higher amount. Therefore, from the chemical‐physical point of view, diverse characteristics emerged when analyzing the two materials because of both the type and the extent of grafted moieties. The main discrepancies were detected in terms of surface zeta potential values and thermal behavior: Cell‐SH showed a more negative surface charge with a higher Z potential and a more complex thermic degradation profile, probably related to the presence of a layer of condensed polysiloxanes.

Both the prepared materials were tested to treat ultrapure water and real water samples spiked with increasing Hg(II) concentrations and the results were compared with those obtained with the pristine cellulose.

The sulfur‐containing material has proven to be the most effective adsorbent: from isotherm calculations, the maximum adsorption capacities were 44 mg g^−1^ and 48 mg g^−1^ in ultrapure and real spiked water, respectively. Furthermore, we demonstrated that 3 mg mL^−1^ of this material is a sufficient quantity to effectively treat a real water sample contaminated with a low amount of mercury, obtaining such removal as to bring its concentration within European legal limits for human usage water (1 μg L^−1^).

In the future, this work can be implemented by widening the application range to other contaminants and other water matrixes, also evaluating the possibility of reusing the materials. As a further perspective, the functionalization of cellulose to introduce amino and thiol groups can be carried out by using more sustainable agents such as amino acids and the production of cellulose itself can be conducted with greener methodologies, as well.

## 4 Experimental Section

4.1

4.1.1

##### Materials

All chemical reagents were in analytical grade and used without further purification. Mercury chloride (HgCl_2_), sodium hydroxide (NaOH), sodium dihydrogen phosphate (H_2_NaPO_4_·H_2_O), (3‐aminopropyl)triethoxysilane (APTES), (3‐mercaptopropyl)tri‐methoxysilane (MPTMS), ethanol, and acetone were purchased from Merck, while hydrochloric acid (HCl) from Carlo Erba. Fresh yellow soybean (*Glycine max*) seeds were purchased from Del Prete s.r.l. (Fondi, LT, Italy) and stored at room temperature before use.

##### Isolation of Cellulose from Soybean Hulls

Cellulose was isolated from deproteinized soybean hulls. Proteins were removed by means of a procedure used for the extraction of soybean peroxidase (SBP).^[^ ^85^, ^86^ ^]^ In brief, soybean seeds were peeled, added to phosphate buffer (0.025 M, pH 7), and stirred for 2 h at room temperature. Then, the hulls were separated from the solution by filtration with cotton gauze and subjected again to the same treatment until the filtrate responded negatively to the enzymatic activity test for SBP.^[^ ^87^ ^]^ The hulls were successively dried at room temperature and ground in an agate mortar. The biomass was treated with a 2% w/v sodium hydroxide solution (solid–liquid ratio 1:10) for 2 h at 80 °C, then washed with distilled water up to a neutral pH value, and dried at 60 °C. Successively, the pretreated pulp was subjected to acid hydrolysis with 1 M HCl (solid‐liquid ratio 1:10) at 80 °C for 2 h, then washed with distilled water up to a neutral pH, and dried at 60 °C. Finally, the pulp was treated once again with 2% w/v NaOH solution (solid–liquid ratio 1:10), washed, and dried again to obtain the final product.^[^ ^86^, ^88^, ^89^ ^]^

##### Cellulose Functionalization

Two functionalized cellulose samples, namely Cell‐NH_2_ and Cell‐SH, were synthesized and tested for mercury adsorption.

For Cell‐NH_2_ synthesis, an APTES‐functionalization method proposed by Tummino et al.^[^ ^32^ ^]^ was employed with minor modifications. In brief, 1 g of cellulose was dispersed in 20 mL of ethanol and 3 mL of APTES was added. The mixture was then refluxed at room temperature for 24 h under stirring, filtered, washed with distilled water, and dried at 100 °C.

For Cell‐SH preparation, the MPTMS‐functionalization procedure proposed by Beaumont et al.^[^ ^37^ ^]^ was used: 1 g of cellulose was dispersed in 100 mL of distilled water, and then 2 mL of HCl 0.5 M and 3.2 mL of MPTMS were added. The mixture was left under stirring for 30 min and then 4 mL of NaOH 0.5 M was added. After stirring 150 min at room temperature, the sample was filtered and washed with acetone and distilled water to remove the unreacted MPTMS and finally dried.

##### Cellulose Sample Characterization

Elemental analysis (C, N, S, H) was performed on both pristine cellulose and synthesized samples (Cell‐NH_2_ and Cell‐SH) with a FLASH 2000 instrument equipped with a thermal conductivity detector. Nitrogen and sulfur content was used to evaluate the percentage of functionalization of hull‐derived cellulose and the amount of grafted silane (mmol g^−1^) was calculated accordingly.

ATR‐FTIR spectra of hull‐derived cellulose, Cell‐NH_2_, and Cell‐SH samples were recorded using a Spectrum Two UATR (PerkinElmer) instrument in the range 4000–600 cm^−1^.

The morphology of the materials and the element distribution were studied using a FE‐SEM Tescan S9000G instrument with a Schottky emission source and equipped with the Ultim Max detector (Oxford, UK) for microanalysis. AZTEC software was employed for data collection.

The thermal behavior of the samples was investigated by TGA and DSC. For TGA analyses (Mettler Toledo TGA‐DSC 1, Schwerzenbach, Switzerland), the sample (10 mg) was heated from room temperature to 100 °C, left at this temperature for 30 min, and then heated to 1000 °C at a rate of 10 °C min^−1^ under 30 mL min^−1^ of nitrogen flux.

DTG was used to identify the temperature of maximum mass‐loss rates. DSC was carried out with DSC equipment (Mettler Toledo 821e, Schwerzenbach, Switzerland) calibrated by an indium standard. The calorimeter cell was flushed with 100 mL min^−1^ nitrogen. The run was performed from 30 to 500 °C, at the heating rate of 10 °C min^−1^, and the mass sample was about 5 mg. Data processing was conducted using the STARe Software.

Nitrogen volumetric adsorption–desorption isotherms for specific surface area determination were obtained in a Micromeritics TRISTAR 3000 at a temperature of −196 °C. A degasification step of 8 h in nitrogen was performed at 150 °C for all samples. The surface area was calculated using the BET equation in the relative pressure range of 0.05–0.20.^[^ ^90^ ^]^

The Zeta potential of the cellulose‐based samples was determined using A NanoPlus DLS Zeta Potential from Micromeritics. Aqueous suspensions were prepared with a mass‐to‐volume ratio of 0.5 mg mL^−1^ at pH values in the range of 2–9 by adjusting the pH with HCl 0.1 M and NaOH 0.1 M solutions.

##### Real Water Sampling and Characterization

Real water from trout aquaculture production was collected in the farm *Società Agricola San Biagio Delia Revelli* located in the north of Italy (12084 Mondovì, Cuneo). Water was sampled in amber glass bottles, filtered with 70 mm glass microfibers filters (VWR), and stored at 4 °C. The sampled water was analyzed to determine the main anionic species (Ionic chromatography, IC Metrohm Eco 925), metals (Inductively Coupled Plasma Atomic Emission Spectrometry, Agilent 5800 ICP‐OES), and total organic carbon content (TOC) (Shimadzu TOC‐5000 analyzer).

##### Mercury Adsorption Tests

Mercury adsorption experiments were performed to evaluate the effect of Hg(II) initial concentration, solutions’ pH, and the role of the water matrix on the adsorption behavior of the synthesized materials.

Mercury stock solutions were prepared starting from mercuric chloride (HgCl_2_). The concentration of Hg(II) in the aqueous solutions throughout all the experiments was determined by cold vapor atomic fluorescence spectroscopy using a PSA Analytical Millennium Merlin instrument. The analyses were performed following the EN 13506 standard procedure.

Cellulose‐based adsorbent dose was set at 1 mg mL^−1^ if not otherwise specified. Kinetic experiments were performed in a 20 mg L^−1^ Hg(II) solution to select the contact time needed to reach the equilibrium conditions.

The amount of mercury adsorbed at each time, *q*_t_ (mg g^−1^), was calculated according to the Equation (1)

(1) $$q_{t}=\frac{\left(C_{0}-C_{t}\right)\cdot V}{W}$$

where *C*_0_ (mg L^−1^) is the initial concentration of Hg(II), *C*_t_ (mg L^−1^) is the concentration after the adsorption experiment, *V* (mL) is the volume of the solution, and *W* (g) is the mass of the adsorbent.

The pseudo‐first‐ and pseudo‐second‐order kinetic models have been used to fit the kinetic experimental data according to the linearized Equation (2) and (3)^[^ ^91^ ^]^

(2) $$\text{ln}(q_{e}-q_{t})=\text{ln}\left(q_{e}\right)-k_{1}t$$

(3) $$\frac{t}{q_{t}}=\frac{1}{k_{2}\times q_{e}^{2}}+\frac{t}{q_{e}}$$

where *q*_e_ (mg g^−1^) and *q*_t_ (mg g^−1^) are the amount of mercury adsorbed at equilibrium and at each time respectively, *k*_1_ (min^−1^) and *k*_2_ (g mg^−1^ min^−1^) are the pseudo‐first‐ and pseudo‐second‐order constants.

The equilibrium isotherms were built at 20 °C and at natural pH 5 and were obtained by changing mercury concentration (1‐200 mg L^−1^). maintaing the adsorbent dose constant. Such isotherms were mainly described using the Langmuir and Freundlich models. The first one considers the adsorption as chemisorption with the formation of a monolayer of adsorbate^[^ ^92^ ^]^ and it is described by Equation (4)

(4) $$q_{e}=\frac{q_{m}\cdot K_{L}\cdot C_{e}}{1+K_{L}\cdot C_{e}}$$

where *q*_m_ (mg L^−1^) is the maximum adsorption capacity and *K*_L_ (L mg^−1^) is the Langmuir constant, which is related to the energy of the adsorption process.

The isotherm parameters were calculated using the linearized Equation (5)

(5) $$\frac{C_{e}}{q_{e}}=\frac{1}{q_{m}}\cdot C_{e}+\frac{1}{K_{L}\cdot q_{m}}$$

The separation factor *R*_L_ was also calculated using Equation (6), as an index of the reversibility or irreversibility of the adsorption process

(6) $$R_{L}=\frac{1}{1+K_{L}\cdot q_{m}}$$

When *R*_L_ = 0, it means that the isotherm is irreversible, and when *R*_L_ = 1, it indicates a linear isotherm. Moreover, if 0 < *R*_L_ < 1, the isotherm is favorable, whereas *R*_L_ > 1 indicates an unfavorable isotherm.^[^ ^93^ ^]^

On the other hand, the Freundlich model^[^ ^94^ ^]^ considers the possible formation of multilayers of adsorbate or different surface energies for the monolayer in heterogeneous materials. This model is described by Equation (7)

(7) $$q_{e}=K_{F}\cdot C_{e}^{1/n}$$

where *C*_e_ (mg L^−1^) is the Hg(II) equilibrium concentration, and *K*_F_ and *n* are the Freundlich constant and the heterogeneity factor, respectively. The value of the 1/*n* factor indicates if the isotherm is favorable (0 < 1/*n* < 1), unfavorable (1/*n* > 1), or irreversible (1/*n* = 0).^[^ ^95^ ^]^

Isotherm parameters were calculated using the linearized Equation (8)

(8) $$\text{ln}(q_{e})=\text{ln}(K_{F})+\frac{1}{n}\text{ln}(C_{e})$$

Finally, additional adsorption runs were carried out in real water from aquaculture production spiked with Hg(II) (1 mg L^−1^).

## Conflict of Interest

The authors declare no conflict of interest.

## Author Contributions

**Monica Rigoletto**: data curation (equal); investigation (equal); writing—original draft (equal). **María Rapp**: data curation (equal); investigation (equal); **Amaya Arencibia**: conceptualization (equal); writing—review editing (equal). **María‐José López‐Muñoz**: conceptualization (equal); writing—review editing (equal). **Maria Laura Tummino**: investigation (equal); writing—review editing (equal). **Nieves Fernández de Paz**: investigation (supporting); resources (supporting). **Enzo Laurenti**: conceptualization (equal); writing—original draft (equal); writing—review editing (equal).

## Data Availability

The data that support the findings of this study are available from the corresponding author upon reasonable request.
